# Exact Enumeration Approach to Estimate the Theta Temperature of Interacting Self-Avoiding Walks on the Simple Cubic Lattice

**DOI:** 10.3390/polym14214536

**Published:** 2022-10-26

**Authors:** Sing-Shuo Huang, Yu-Hsin Hsieh, Chi-Ning Chen

**Affiliations:** Department of Physics, National Dong-Hwa University, Hualien 974, Taiwan

**Keywords:** collapse transition, interacting self-avoiding walks, exact enumeration, Bulirsch–Stoer extrapolation

## Abstract

We compute the exact root-mean-square end-to-end distance of the interacting self-avoiding walk (ISAW) up to 27 steps on the simple cubic lattice. These data are used to construct a fixed point equation to estimate the theta temperature of the collapse transition of the ISAW. With the Bulirsch–Stoer extrapolation method, we obtain accurate results that can be compared with large-scale long-chain simulations. The free parameter ω in extrapolation is precisely determined using a parity property of the ISAW. The systematic improvement of this approach is feasible by adopting the combination of exact enumeration and multicanonical simulations.

## 1. Introduction

With the rapid development of computer hardware and software, computational approaches [1,2,3] are increasingly important in polymer science. Among various computational methods, exact enumeration is a very primitive technique. Almost four decades passed since W.J.C. Orr [4] studied the equilibrium properties of a single polymer chain at dilute solution by exact enumeration. In this paper, we show that exact enumeration can already produce quantitative results as accurate as those of large-scale simulations. We focus on the interacting self-avoiding walk (ISAW) on the simple cubic lattice [5,6,7,8,9,10]. The ISAW model is a very basic polymer model that serves as the framework of most lattice protein models [11,12].

An ISAW is a self-avoiding walk with attraction between monomers. The energy of an ISAW chain is defined as *m*(-ε), where *m* is the number of nonconsecutive nearest-neighbor contacts, and -ε is the attractive contact energy between two monomers. The canonical partition function of a *N*-step ISAW is

(1) $$Z_{N}\left(x\right)=\sum_{\mathrm{all}\;\mathrm{ISAW}\mathrm{conf}.}e^{-E/k_{{}_{B}}T}=\sum_{\mathrm{all}\;\mathrm{ISAW}\mathrm{conf}.}{\left(e^{\varepsilon /k_{{}_{B}}T}\right)}^{m}=\sum_{m=0}^{M}c_{m}\;x^{m}$$

where $x=e^{\varepsilon /k_{{}_{B}}T}$, $c_{m}$ is the number of ISAW configurations with *m* contacts, and *M* is the maximal number of contacts. Here, ε and $k_{{}_{B}}$ can be set to 1 by adjusting the units. Another important function related to the square of the end-to-end distance is defined as follows:

(2) $$R_{N}^{2}\left(x\right)=\sum_{m=0}^{M}\sum_{R^{2}=1}^{N^{2}}R^{2}\;c_{m,R^{2}}\;x^{m}$$

where $R^{2}$ is the square end-to-end distance, $c_{m,R^{2}}$ is the number of ISAW configurations with *m* contacts and square end-to-end distance $R^{2}$, and $c_{m,R^{2}}$ satisfies $\sum_{R^{2}=1}^{N^{2}}\;c_{m,R^{2}}=c_{m}$. Both $Z_{N}\left(x\right)$ and $R_{N}^{2}\left(x\right)$ are polynomials of *x* with positive integer coefficients. The root-mean-square end-to-end distance at a certain temperature can be expressed as follows:

(3) $$R_{N}\left(T\right)=\sqrt{\frac{R_{N}^{2}\left(e^{1/T}\right)}{Z_{N}\left(e^{1/T}\right)}}$$

**Figure 1:**
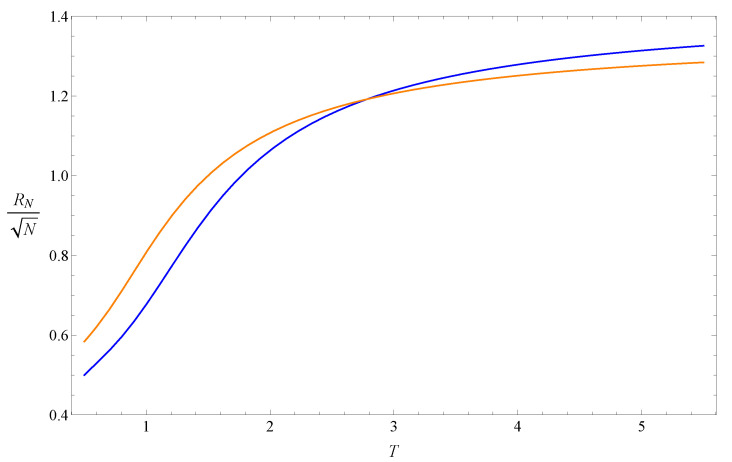
Curves of $R_{N}\left(T\right)$ defined in Equation (3) and normalized by $\sqrt{N}$ with N=26 (blue) and N=16 (orange).

Two examples of the normalized end-to-end function $R_{N}\left(T\right)/\sqrt{N}$ are shown in Figure 1. Both of them are increasing functions, and the slope of the function with larger *N* is also larger around the intersection point.

A polymer chain with attraction between monomers undergoes a collapse transition at theta temperature $T_{\theta }$. $R_{N}\left(T\right)$ has different scaling behaviors at different temperature regions [13]:

(4) $$\begin{array}{ccccc} \; & \; & & N^{\nu_{{}_{saw}}}, & T>T_{\theta }\\ R_{N}\left(T\right) & ∼ & \; & N^{\nu_{\theta }}f\left(N^{\phi }\left(T-T_{\theta }\right)\right), & T∼T_{\theta }\\ \; & \; & & N^{1/d}, & T<T_{\theta } \end{array}$$

In three dimensions, $\nu_{\theta }$ and ϕ can be determined by the mean-field theory to both be 1/2. $\nu_{{}_{saw}}$ can be determined with great accuracy by simulation to be 0.587597(7) [14].

The precise estimation of the theta temperature of the ISAW on the simple cubic lattice came from large-scale simulations [15,16,17,18,19,20]. P. Grassberger [17] proposed the well-known pruned-enriched Rosenbluth method (PERM) on the basis of the Rosenbluth–Rosenbluth method and the idea of enrichment. For free chains with *N* = 10,000, the best estimate of the theta temperature was 3.717(3). The recursive sampling algorithm used by P. Grassberger and R. Hegger [15] was a previous version, and the estimated theta temperature was 3.721(6) with *N* = 5000. H. Frauenkron and P. Grassberger [18] used the PERM to simulate polymer solutions with *N* = 2048 and obtained an estimated theta temperature of 3.717(2). T. Vogel et al. [20] used the new PERM with simple sampling up to *N* = 32,000 and performed a scaling analysis to obtain an estimate of 3.72(1). The above are all chain-growth methods. Tesi et al. [16] used two Markov chain-sampling methods, the multiple Markov chain method and umbrella sampling, to obtain an estimated theta temperature of 3.62(8) with *N* = 1600. Yan et al. [19] used the expanded grand-canonical ensemble simulation for polymer solutions with *N* = 16,000 and obtained an estimate of 3.71(1). In general, Monte Carlo methods must be able to generate unbiased samples and overcome the trapping problems. Exact enumeration, in contrast, is much clearer and simpler. The only challenge with exact enumeration is how to count the total number of larger systems. This is more of a computational problem than a theoretical problem. The solution to this problem benefits directly from the rapid development of computer hardware and software.

## 2. Method

The computational methods used in this paper are the exact enumeration algorithm to count the total number of ISAW configurations, and the Bulirsch–Stoer algorithm to extrapolate the finite-size data.

### 2.1. Exact Enumeration

To determine $c_{m}$ and $c_{m,R^{2}}$ in Equations (1) and (2), we used a direct counting algorithm that we developed [10] to exhaustively enumerate all configurations of an ISAW chain on the simple cubic lattice. The original goal of this algorithm was to generate enough typical sequences for the 27-mers [21] to study the relationship between protein sequences and structures. Using this algorithm to count all configurations (not just the ground states) of a protein sequence with 27 monomers on the simple cubic lattice now only takes the order of days. The algorithm includes not only direct counting but also reduction in the degrees of freedom in the beginning and final stages. These procedures lead to a significant reduction in computation time.

**Figure 2:**
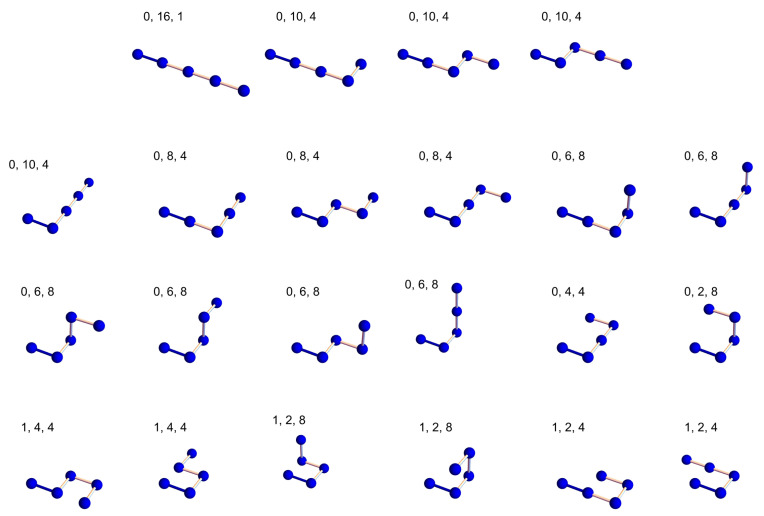
All configurations of a 4-step ISAW on the simple cubic lattice.

In the following, we explain some details of the counting process. Figure 2 shows all 22 representative configurations of a four-step ISAW on the simple cubic lattice. The convention is the first monomer being placed at the origin and the second monomer at (1, 0, 0), which fixes the first direction (the darker bond in every configuration). The third monomer has four or five possible directions to choose from, and so on. The three numbers above each configuration in Figure 2 are the number of contacts *m*, the square end-to-end distance $R^{2}$, and degeneracy coming from the symmetry of the next direction taken. If the numbers are 0, 10, and 4, variable $c_{0,10}$ would be incremented by 4 in the program.

Let us consider the six configurations in the last row of Figure 2 as an example. They are configurations with one contact, and they contribute to the coefficients of the linear terms in $Z_{4}\left(x\right)$ and $R_{4}^{2}\left(x\right)$. $c_{1}=4+4+8+8+4+4=32$. $c_{1,2}=8+8+4+4=24$. $c_{1,4}=4+4=8$. The coefficient $\sum_{R^{2}}R^{2}\;c_{1,R^{2}}$=2×24+4×8=80. The final results are $Z_{4}\left(x\right)=32x+89$ and $R_{4}^{2}\left(x\right)=80x+592$. The root-mean-square end-to-end distance is thus

(5) $$R_{4}\left(T\right)=\sqrt{\frac{80e^{1/T}+592}{32e^{1/T}+89}}$$

The direct counting algorithm has the advantage of easily integrating different ideas and techniques. One straightforward parallel implementation for this direct counting algorithm runs 22 jobs with 22 initial configurations shown in Figure 2. The number of jobs can be flexibly adjusted by choosing a different number of initial configurations depending on how many computer cores are available. Lastly, all results are collected and summed up to be the exact coefficients of $Z_{N}\left(x\right)$ and $R_{N}^{2}\left(x\right)$. All counting jobs can be completed within a few weeks using a small PC cluster.

### 2.2. Bulirsch–Stoer Extrapolation

The Bulirsch–Stoer algorithm [22,23,24,25] may be the most powerful extrapolation method in existence. Its idea was borrowed from recursive algorithms such as the Richardson and Neville algorithms, but the result is much more general. In this subsection, we briefly introduce the Bulirsch–Stoer algorithm and explain how to use its main formula. The detailed derivation and proof can be found in [25].

In this paper, the data of the finite-size theta temperature of the ISAW needs to be extrapolated. Suppose its finite-size scaling behavior can be described by power-law functions:

(6) $$T\left(N\right)=T_{\theta }+a_{1}N^{-\phi }+a_{2}N^{-\omega_{2}}+a_{3}N^{-\omega_{3}}+\ldots$$

where *N* is the number of steps of the ISAW chain, ϕ is the leading exponent, $0<\phi <\omega_{2}<\omega_{3}<\ldots$, and T(N) is the finite-size scaling function. We may first consider the approximation that ϕ=ω, $\omega_{2}=2\omega$, $\omega_{3}=3\omega$, …. Define $h=N^{-\omega }$, then $T\left(h\right)=T_{\theta }+a_{1}h+a_{2}h^{2}+\ldots$ becomes a polynomial function, to which the Neville algorithm can be applied. Below, we use five data points, $\{\left(h_{i},T\left(h_{i}\right)\right)\},i=0,\ldots ,4$, as an example to illustrate explicitly the basic procedures of the Neville and Bulirsch–Stoer algorithms, while general formulas are also provided. First, a triangular 5×5 matrix $\left(T_{i,j}\right)$ is prepared, and its first column is filled with $\{T\left(h_{i}\right)\},i=0,\ldots ,4$:

$$\left(\begin{array}{ccccc} T_{0,0}=T\left(h_{0}\right) & T_{0,1} & T_{0,2} & T_{0,3} & T_{0,4}\\ T_{1,0}=T\left(h_{1}\right) & T_{1,1} & T_{1,2} & T_{1,3} & 0\\ T_{2,0}=T\left(h_{2}\right) & T_{2,1} & T_{2,2} & 0 & 0\\ T_{3,0}=T\left(h_{3}\right) & T_{3,1} & 0 & 0 & 0\\ T_{4,0}=T\left(h_{4}\right) & 0 & 0 & 0 & 0 \end{array}\right)$$

This matrix can also be expressed as a lower triangular matrix. In this case, the indices in the formulas need a little adjustment. The elements of the second column are then defined as the first-degree Lagrangian polynomials for the data in the first column. For example,

(7) $$T_{0,1}=\frac{h-h_{1}}{h_{0}-h_{1}}\;T_{0,0}+\frac{h-h_{0}}{h_{1}-h_{0}}\;T_{1,0}$$

$T_{0,1}$ obviously passes through $\left(h_{0},T\left(h_{0}\right)\right)$ and $\left(h_{1},T\left(h_{1}\right)\right)$. Neville noticed that the formula of the same form can be used in the third column. For example,

(8) $$T_{0,2}=\frac{h-h_{2}}{h_{0}-h_{2}}\;T_{0,1}+\frac{h-h_{0}}{h_{2}-h_{0}}\;T_{1,1}$$

$T_{0,2}$ can be shown by straightforward algebra to be the second-degree Lagrangian polynomial passing through $\left(h_{0},T\left(h_{0}\right)\right)$, $\left(h_{1},T\left(h_{1}\right)\right)$, and $\left(h_{2},T\left(h_{2}\right)\right)$. The same is true for all following columns. Thus, the general formula is:

(9) $$\begin{array}{cc} T_{i,j}= & \frac{h-h_{i+j}}{h_{i}-h_{i+j}}\;T_{i,j-1}+\frac{h-h_{i}}{h_{i+j}-h_{i}}\;T_{i+1,j-1}\\ = & T_{i+1,j-1}+\frac{T_{i+1,j-1}-T_{i,j-1}}{\left(\frac{h-h_{i}}{h-h_{i+j}}\right)-1} \end{array}$$

Equation (9) is the main formula of the Neville algorithm. The final output for this five-point example is $T_{0,4}$, the fourth-degree Lagrange polynomial passing through all five data points. It is an extrapolation function that can be used to approximate the finite-size scaling function T(h). Neville showed that the Lagrangian polynomials can be generated in such an iterated way. The Neville algorithm is very efficient in evaluating function values for interpolation or extrapolation.

Bulirsch and Stoer inserted an additional term: $\left(1-\left(T_{i+1,j-1}-T_{i,j-1}\right)/\left(T_{i+1,j-1}-T_{i,j-2}\right)\right)$, in the denominator in Equation (9):

(10) $$T_{i,j}=T_{i+1,j-1}+\frac{T_{i+1,j-1}-T_{i,j-1}}{\left(\frac{h-h_{i}}{h-h_{i+j}}\right)\left(1-\frac{T_{i+1,j-1}-T_{i,j-1}}{T_{i+1,j-1}-T_{i,j-2}}\right)-1}$$

The appearance of $T_{i,j-2}$ requires $\{T_{i,-1}\},i=0,\ldots ,4$ to be defined first. They can be set to zero in the beginning. The effect of the additional term is that $T_{0,4}$ now becomes a rational function P(h)/Q(h), where both P(h) and Q(h) are polynomials. This rational function also passes through all the data points and is another extrapolation function that can be used to approximate the finite-size scaling function T(h). Since T(h) is actually not a polynomial, it is expected that the more general rational functions are more suitable to model T(h) than polynomials. Our extrapolation results also show that the Bulirsch–Stoer algorithm is always more accurate than the Neville algorithm. More details on the properties of such rational functions appearing in Bulirsch–Stoer extrapolation can be found in [25].

In Equation (6), $T_{\theta }$ is reached as N→∞, or h→0. Substituting h=0 and $h_{i}=N_{i}^{-\omega }$ into Equation (10),

(11) $$T_{i,j}=T_{i+1,j-1}+\frac{T_{i+1,j-1}-T_{i,j-1}}{{\left(\frac{N_{i+j}}{N_{i}}\right)}^{\omega }\left(1-\frac{T_{i+1,j-1}-T_{i,j-1}}{T_{i+1,j-1}-T_{i,j-2}}\right)-1}$$

Equation (11) is the main formula used in this paper to extrapolate the finite-size theta temperatures. Now $T_{0,4}$ is the extrapolation estimation of the theta temperature with five data points. $T_{0,3}$ and $T_{1,3}$ are also the estimations of the theta temperature but with only four data points used. They are less accurate than $T_{0,4}$, while their difference can serve as an estimate of the error of $T_{0,4}$. A simple argument is as follows. Suppose $T_{0,3}=T_{\theta }\pm \sigma_{0,3}$, $T_{1,3}=T_{\theta }\pm \sigma_{1,3}$, and $T_{0,4}=T_{\theta }\pm \sigma_{0,4}$, where $\sigma_{0,3}$, $\sigma_{1,3}$, and $\sigma_{0,4}$ are the statistical errors of $T_{0,3}$, $T_{1,3}$, and $T_{0,4}$. $\left(T_{0,3}-T_{1,3}\right)$ can be expressed as $0\pm \sqrt{\sigma_{0,3}^{2}+\sigma_{1,3}^{2}}$. Its range is larger than $\pm \sigma_{0,4}$, since both $\sigma_{0,3}$ and $\sigma_{1,3}$ are larger than $\sigma_{0,4}$. Thus, $|T_{0,3}-T_{1,3}|$ can roughly play the role of $\sigma_{0,4}$. Although there are actually no statistics for $T_{i,j}$, this error estimation is appropriate for most testing examples where the answers are known. The premise is that $T_{0,3}$, $T_{1,3}$, and $T_{0,4}$ should be close to each other to indicate that the extrapolation values have entered a stable region. In this paper, we adopt the following definition of the error of $T_{0,j}$:

(12) $$\epsilon_{0,j}\equiv \left|T_{0,j-1}-T_{1,j-1}\right|$$

In Equation (11), ω is a free parameter. ω does not need to be equal to ϕ, but it won’t be very different either. Each ω is associated with an extrapolation function that passes through all the data points. A different ω results in a different extrapolation value. It is, thus, very important to choose ω carefully. A reasonable choice of ω is the one that minimizes the error defined in Equation (12). In this paper, we use the parity property of the ISAW on the simple cubic lattice to more precisely determine ω.

## 3. Result

On the basis of Equation (4), the solution $T^{*}$ of the following equation is an estimation of theta temperature $T_{\theta }$:

(13) $$\frac{R_{N}\left(T^{*}\right)}{\sqrt{N}}=\frac{R_{N^{\prime }}\left(T^{*}\right)}{\sqrt{N^{\prime }}}$$

The choice of *N* and $N^{\prime }$ should be both odd or both even. The random walks on the square or simple cubic lattice are naturally divided into two groups, namely, random walks with the even number of steps and the odd number of steps. On the simple cubic lattice, even-step walks only stop at the point (i,j,k) with even (i+j+k), and odd-step walks only stop at the point with odd (i+j+k). For this reason, random walks with the same parity are more similar to each other. When *N* and $N^{\prime }$ are closer, the two walks would also be more similar, and Equation (13) would give more accurate estimations. Our numerical results confirmed this expectation. Therefore, we only discuss the case of $N-N^{\prime }=2$ in this paper. Equation (13) becomes

(14) $$\frac{R_{N+1}\left(T^{*}\left(N\right)\right)}{\sqrt{N+1}}=\frac{R_{N-1}\left(T^{*}\left(N\right)\right)}{\sqrt{N-1}}$$

**Figure 3:**
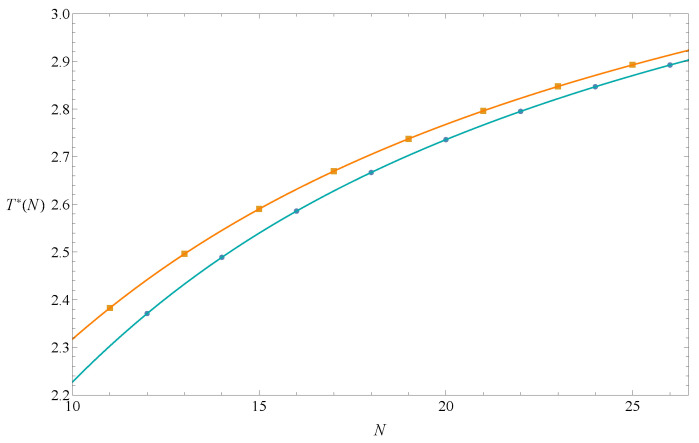
$T^{*}\left(N\right)$ determined by Equation (14) for odd and even *N*. The two lines that pass through eight odd data points and eight even data points are seventh-degree Lagrangian polynomial graphs.

We generated square end-to-end functions $R_{N}^{2}\left(x\right)$ for 10≤N≤27 (listed in Appendix A), and used them and Equation (14) to calculate $T^{*}\left(N\right)$. The results were divided into two groups according to parity and are listed in Table 1. Figure 3 also shows that the data points were clearly divided into two groups. The two lines passing through the data points are seventh-degree Lagrangian polynomials. They will merge as N→∞. The estimation of the theta temperature could be reasonably set as $(T_{{}_{I}}+T_{{}_{II}})/2$≡ ($T^{*}\left(N_{\mathrm{odd}}\to \infty \right)+T^{*}\left(N_{\mathrm{even}}\to \infty \right))/2$. Its error is defined as $\sqrt{\epsilon_{{}_{I}}^{2}+\epsilon_{{}_{II}}^{2}}/2$, where $\epsilon_{{}_{I}}$ and $\epsilon_{{}_{II}}$ are errors of $T_{{}_{I}}$ and $T_{{}_{II}}$ (Equation (12)) in the Bulirsch–Stoer extrapolation method. Two extrapolations need to be performed here, and the chosen ω needs to make both $\epsilon_{{}_{I}}$ and $\epsilon_{{}_{II}}$ smaller. Since there are two constraints, the extrapolation value would be less biased.

To determine an optimal ω, a wide range of ω is scanned first to find the region with small errors. The adjacent area is then zoomed in until the results do not change. Table 2 lists the estimations and errors of the theta temperature in the range of 0.814≤ω≤0.825. The error is minimal as ω=0.820, so the best estimation was $T_{\theta }=3.709\left(2\right)$. This result is consistent with the long-chain results of large-scale simulations [15,16,17,18,19,20]. We also used the Neville algorithm (Equation (9)) instead of the Bulirsch–Stoer algorithm to extrapolate the same data. The best estimation was $T_{\theta }=3.501\left(4\right)$ with ω=1.404. For all data that we examined, the results of the Bulirsch-Stoer algorithm were always better than the results of the Neville algorithm. Extrapolation with rational functions is expected to be better than extrapolation with polynomials unless the finite-size scaling function is inherently a polynomial.

We also considered a correction term predicted by the field-theoretic renormalization group calculation [26]:

(15) $$\frac{R_{N+1}(T^{*}\left(N\right)}{\sqrt{\left(N+1\right)\left(1-\frac{37}{363\;\log(N+1)}\right)}}=\frac{R_{N-1}(T^{*}\left(N\right)}{\sqrt{\left(N-1\right)\left(1-\frac{37}{363\;\log(N-1)}\right)}}$$

This correction term is small and may not agree with the simulations [15,17]. We followed the same procedure as above to calculate $T^{*}\left(N\right)$ with Equation (15), and list the results in Table 3 for comparison.

The best estimation from the data in Table 3 was $T_{\theta }=3.713\left(2\right)$ with ω=0.8617. Since $T^{*}\left(N\right)$ listed in Table 3 was closer to the value of the “real” $T_{\theta }$ than those in Table 1, the extrapolation result seemed to be also improved.

We may use the same procedure to calculate other quantities. For example, we can verify the value of the crossing exponent ϕ in Equation (4) in the following way.

(16) $$\frac{d\;\log R_{N}}{dT}{|}_{T_{\theta }}∼N^{\phi }$$

(17) $$\phi \left(N\right)=\frac{\log\left(\frac{d\;\log R_{N+1}}{dT}{|}_{T_{\theta }}/\frac{d\;\log R_{N-1}}{dT}{|}_{T_{\theta }}\right)}{\log((N+1)/(N-1))}$$

Equation (17) is a ratio method. We took 3.713 as the value of $T_{\theta }$. The results are listed in Table 4. The best estimation of ϕ was 0.531(9) with ω=1.76, while the exact value is 1/2. This result is not as accurate as that of theta temperature estimation. The correction term seems to be important when computing critical exponents. We have tried to include a correction term and obtained much more accurate ϕ results. However, since this paper mainly regards the efficiency of direct computation, we do not discuss the effect of correction further.

## 4. Discussion

The longest ISAW chain used in the calculation of this paper is the 27-step walk. The total number of its configurations is huge: 431,645,810,810,533,429 $∼4\times 10^{17}$. From such a large number and others, we obtained an accurate estimate of the theta temperature of ISAW on the simple cubic lattice. Nevertheless, a 27-step ISAW is very short in three dimensions. The finite-size effect should be obvious. There are two reasons why short chains can still give accurate results. The first reason is the careful determination of the free parameter ω in the Bulirsch–Stoer algorithm. For ISAWs on the simple cubic lattice, we could just take advantage of their natural division into odd and even groups. There are two constraints on the choice of ω, resulting in more stable and accurate extrapolation results. This experience may be extended to other problems where data points are not smooth. The way of classifying the data points and combining the extrapolation results could work. If only one series of data is extrapolated, unless the data points are very smooth, the ω corresponding to the minimal error does not necessarily produce extrapolation values very close to the true answer.

Another reason is that Equation (13) may be regarded as a fixed-point equation of the real-space renormalization group transformation for the coarse-graining process, where each *N*-step segment of the ISAW is replaced by a $N^{\prime }$-step segment ($N^{\prime }<N$). In this view, *N* and $N^{\prime }$ are the block sizes of the transformation and not the length of the whole ISAW chain. The finite-size effect could, therefore, be less obvious. Equation (13) can be rewritten as the equation to transform *T* to $T^{\prime }$: $R_{N^{\prime }}\left(T^{\prime }\right)/\sqrt{N^{\prime }}=R_{N}\left(T\right)/\sqrt{N}$, which will drive the temperature away from the intersectional temperature $T^{*}$. Figure 1 shows this feature: if $T>T^{*}$, $T^{\prime }$ is larger than *T*, and $T<T^{*}$, $T^{\prime }$ is smaller than *T*. A simple explanation is that, if $T>T^{*}$, $R_{N^{\prime }}\left(T\right)/\sqrt{N^{\prime }}∼$${N^{\prime }}^{\nu_{saw}-1/2}<N^{\nu_{saw}-1/2}$$∼R_{N}\left(T\right)/\sqrt{N}$, so $T^{\prime }$ needs to be larger to balance the transformation equation. A similar logic could be used for the case of $T<T^{*}$. Only in the critical region around $T^{*}$, $T^{\prime }\approx T$, and the normalized end-to-end distances of the two ISAW segments with length *N* and $N^{\prime }$ are approximately the same. The larger and closer *N* and $N^{\prime }$ are, the more similar these two segments are, and the transformation equation is more accurate, so that $T^{*}$ is closer to $T_{\theta }$. Traditional real-space renormalization approaches for polymer models usually focus on the variable fugacity, e.g., [27]. The above scenario of the real-space renormalization along the ISAW chain is only for the variable temperature and can be developed in more detail. It is also similar to phenomenological renormalization, e.g., [28], in which the correlation length plays the same role as $R_{N}\left(T\right)$ and can be calculated by the transfer matrix method.

The number of configurations of an *N*-step ISAW grows exponentially with *N*, e.g., $\mu^{N}$ with μ∼4.7 for the simple cubic lattice. From 27 to 30 steps, the number of configurations is increased by about 100 times. To go further, the degree of freedom of the problem must be reduced in some way. The transfer matrix technique is efficient for the counting problem of the two-dimensional ISAW [29,30], but difficult to extend to the three-dimensional case. Matrix methods have been used in polymer science for many years, e.g., [31,32], and deserve further development. The length-doubling method [33,34] reduces the degree of freedom by counting and saving the information of two short walks and then joining them to form a longer walk. It is very feasible for us to use this approach because we had developed a counting algorithm with a similar idea [35]. We counted the number of graphs in the percolation and Potts models by dividing the graphs into two parts (or several layers) and then combining the two parts of the data to obtain the complete result. This algorithm was used to study the partition function zeros of the Potts model on the self-dual lattices [36].

We can push to the limit the size of the system that can be exactly enumerated to obtain more accurate results. However, this direction may become very technical. A more practical approach is to perform the multicanonical simulation [37,38] to count the configurations of longer chains. The multicanonical simulation accumulates the density of states during Monte Carlo sampling and is very different from the Metropolis algorithm, in which sampling is just for averaging. This approach can be called Monte Carlo counting, corresponding to exact counting. Among multicanonical methods, the Wang–Landau algorithm [39] is commonly used because of its concise steps and high efficiency. It has been applied to polymer simulations of various systems, including the ISAW [40]. We have tried to combine the data of medium-length chains by Wang–Landau sampling with the data of short chains by exact enumeration. Although the numbers of configurations from the Wang–Landau method are approximate, they carry information about the longer chains and would stabilize the extrapolation curve around the medium-length region. Therefore, the data from the Wang–Landau method would certainly improve the accuracy of exact enumeration. If there is no need to reach the limit, the total computation time spent on exact enumeration and Wang–Landau sampling together could still be less than that of large-scale simulations. This approach of combining exact counting and Monte Carlo counting might become a practical and general technique in computational science.

In summary, we used exact enumeration and Bulirsch–Stoer extrapolation to obtain an accurate estimate of the theta temperature of the ISAW on the simple cubic lattice. The systematic improvement of this approach by increasing the chain length is achievable. Research in this direction is in progress.

## Figures and Tables

**Table 1:** Estimation of the theta temperature $T^{*}\left(N\right)$ determined by Equation (14).

$N_{\mathrm{odd}}+1/N_{\mathrm{odd}}-1$	$T^{*}\left(N_{\mathrm{odd}}\right)$	$N_{\mathrm{even}}+1/N_{\mathrm{even}}-1$	$T^{*}\left(N_{\mathrm{even}}\right)$
12/10	2.3826	13/11	2.3710
14/12	2.4961	15/13	2.4889
16/14	2.5904	17/15	2.5860
18/16	2.6697	19/17	2.6670
20/18	2.7375	21/19	2.7358
22/20	2.7962	23/21	2.7951
24/22	2.8474	25/23	2.8468
26/24	2.8926	27/25	2.8923

**Table 2:** Extrapolation with different ω for the data listed in Table 1, where $T_{{}_{I}}=T^{*}\left(N_{\mathrm{odd}}\to \infty \right)$, $T_{{}_{II}}=T^{*}\left(N_{\mathrm{even}}\to \infty \right)$ and error $=\sqrt{\epsilon_{{}_{I}}^{2}+\epsilon_{{}_{II}}^{2}}/2$.

ω	$T_{{}_{I}}$	$T_{{}_{\mathrm{II}}}$	$(T_{{}_{I}}+T_{{}_{\mathrm{II}}})/2$	Error
0.814	3.7164	3.7053	3.7109	0.00212
0.815	3.7160	3.7051	3.7106	0.00206
0.816	3.7156	3.7048	3.7102	0.00201
0.817	3.7152	3.7046	3.7099	0.00196
0.818	3.7148	3.7043	3.7096	0.00191
0.819	3.7144	3.7041	3.7093	0.00187
0.820	3.7140	3.7038	3.7089	0.00184
0.821	3.7136	3.7036	3.7086	0.00185
0.822	3.7132	3.7033	3.7083	0.00193
0.823	3.7128	3.7031	3.7080	0.00212
0.824	3.7124	3.7029	3.7076	0.00246
0.825	3.7120	3.7026	3.7073	0.00304

**Table 3:** The estimation of theta temperature $T^{*}\left(N\right)$ determined by Equation (15).

$N_{\mathrm{odd}}+1/N_{\mathrm{odd}}-1$	$T^{*}\left(N_{\mathrm{odd}}\right)$	$N_{\mathrm{even}}+1/N_{\mathrm{even}}-1$	$T^{*}\left(N_{\mathrm{even}}\right)$
12/10	2.5421	13/11	2.5112
14/12	2.6345	15/13	2.6137
16/14	2.7135	17/15	2.6989
18/16	2.7811	19/17	2.7705
20/18	2.8396	21/19	2.8316
22/20	2.8907	23/21	2.8845
24/22	2.9356	25/23	2.9307
26/24	2.9754	27/25	2.9715

**Table 4:** The estimation of crossing exponent ϕ(N) determined by Equation (17).

$N_{\mathrm{odd}}+1/N_{\mathrm{odd}}-1$	$\phi (N_{\mathrm{odd}})$	$N_{\mathrm{even}}+1/N_{\mathrm{even}}-1$	$\phi (N_{\mathrm{even}})$
12/10	0.8944	13/11	0.8107
14/12	0.8350	15/13	0.7753
16/14	0.7936	17/15	0.7485
18/16	0.7628	19/17	0.7271
20/18	0.7387	21/19	0.7097
22/20	0.7192	23/21	0.6951
24/22	0.7032	25/23	0.6826
26/24	0.6896	27/25	0.6719

## Appendix A

Square end-to-end distance function $R_{N}^{2}\left(x\right)$ was used in the calculation of this paper. $Z_{N}\left(x\right)$ can be found in [8,9,10] and references therein. The convention adopted in these references was $Z_{n}\left(x\right)$, where *n* is the number of monomers, i.e., n=N+1.

$$\begin{array}{cc} R_{10}^{2}\left(x\right)= & \;2400x^{7}+19248x^{6}+93872x^{5}+413192x^{4}+1481904x^{3}+3900024x^{2}+8155040x+10627300\\ R_{11}^{2}\left(x\right)= & \;1576x^{9}+60704x^{7}+224980x^{6}+1021784x^{5}+3351332x^{4}+10256736x^{3}+23633256x^{2}\\ + & \;43562608x+49569849\\ R_{12}^{2}\left(x\right)= & \;16416x^{9}+104512x^{8}+633696x^{7}+2545088x^{6}+8501040x^{5}+25078688x^{4}+66255936x^{3}\\ + & \;136926368x^{2}+226106208x+228218912\\ R_{13}^{2}\left(x\right)= & \;70832x^{10}+233976x^{9}+1651200x^{8}+6655072x^{7}+22621780x^{6}+64631184x^{5}+173180832x^{4}\\ + & \;406624624x^{3}+765359388x^{2}+1146376744x+1039365077\\ R_{14}^{2}\left(x\right)= & \;126240x^{11}+770880x^{10}+4287040x^{9}+16977136x^{8}+61503008x^{7}+174549448x^{6}+463709872x^{5}\\ + & \;1124687768x^{4}+2397976592x^{3}+4155302672x^{2}+5700564608x+4690177508\\ R_{15}^{2}\left(x\right)= & \;61888x^{13}+130256x^{12}+2803328x^{11}+11100208x^{10}+47660636x^{9}+161488660x^{8}\\ + & \;490203648x^{7}+1270912460x^{6}+3127566712x^{5}+6973585084x^{4}+13685618032x^{3}\\ + & \;22019032264x^{2}+27886729240x+20998484289\\ R_{16}^{2}\left(x\right)= & \;86496x^{14}+584352x^{13}+6835616x^{12}+31893952x^{11}+132509024x^{10}+442014976x^{9}\\ + & \;1348809664x^{8}+3602285520x^{7}+8811068576x^{6}+20090484976x^{5}+41678485424x^{4}\\ + & \;76017644448x^{3}+114317397088x^{2}+134529227632x+93373103808\\ R_{17}^{2}\left(x\right)= & \;59824x^{16}+3654432x^{14}+16444272x^{13}+95878120x^{12}+372284152x^{11}+1262660332x^{10}\\ + & \;3760878904x^{9}+10200682256x^{8}+25303393620x^{7}+58144426336x^{6}+124206995660x^{5}\\ + & \;241559023440x^{4}+412635057324x^{3}+583175382496x^{2}+641202383920x+412726821809\\ R_{18}^{2}\left(x\right)= & \;526080x^{16}+9127264x^{15}+47511072x^{14}+274455648x^{13}+1050334736x^{12}\\ + & \;3636975824x^{11}+10704576752x^{10}+29166261984x^{9}+72815986408x^{8}\\ + & \;170368948064x^{7}+368862169528x^{6}+743990812816x^{5}+1364475982032x^{4}\\ + & \;2196186178352x^{3}+2930156906688x^{2}+3024187489584x+1814756487380\\ R_{19}^{2}\left(x\right)= & \;5089568x^{17}+20407688x^{16}+167264872x^{15}+790107600x^{14}+3110776832x^{13}\\ + & \;10457227652x^{12}+31269943372x^{11}+84353385216x^{10}+211763826268x^{9}+498888884332x^{8}\\ + & \;1102327917932x^{7}+2266179194088x^{6}+4337209671764x^{5}+7539106381076x^{4}\\ + & \;11490123782816x^{3}+14527782144164x^{2}+14131957951628x+7942249264057\\ R_{20}^{2}\left(x\right)= & \;11639984x^{18}+65635904x^{17}+507007872x^{16}+2267416192x^{15}+9239143104x^{14}\\ + & \;30415994464x^{13}+91479528480x^{12}+247319350256x^{11}+622324223312x^{10}\\ + & \;1470521522480x^{9}+3290722609424x^{8}+6905473586320x^{7}+13547868240384x^{6}\\ + & \;24705002669024x^{5}+40869785392608x^{4}+59219137485824x^{3}\\ + & \;71188679473136x^{2}+65499250792128x+34614203852528\\ R_{21}^{2}\left(x\right)= & \;5345856x^{20}+25080712x^{19}+290885416x^{18}+1506724320x^{17}+7079603104x^{16}\\ + & \;27568255400x^{15}+91164610648x^{14}+270059662696x^{13}+733051434432x^{12}\\ + & \;1847459865932x^{11}+4375541386376x^{10}+9847545657688x^{9}+20987383612124x^{8}\\ + & \;42098460788708x^{7}+79085417264008x^{6}+137902810558716x^{5}+217880054860396x^{4}\\ + & \;301173099741060x^{3}+345209458064260x^{2}+301363357830060x+150290986646877 \end{array}$$

$$\begin{array}{cc} R_{22}^{2}\left(x\right)= & \;14547872x^{21}+69055152x^{20}+919643968x^{19}+4562201232x^{18}+21709174272x^{17}\\ + & \;82149673648x^{16}+273625614720x^{15}+805758842656x^{14}+2187658338032x^{13}+5518095100440x^{12}\\ + & \;13137281565552x^{11}+29647727014584x^{10}+63811855323824x^{9}+130183534083144x^{8}\\ + & \;250645098373808x^{7}+452192377800560x^{6}+756259374539232x^{5}+1144524804204400x^{4}\\ + & \;1513637339689536x^{3}+1658421352720080x^{2}+1377501603623040x+650334446479452\\ R_{23}^{2}\left(x\right)= & \;9266008x^{23}+21596592x^{22}+448526104x^{21}+2884104384x^{20}+15336968872x^{19}\\ + & \;67818998120x^{18}+253485242488x^{17}+831705797144x^{16}+2439956739948x^{15}\\ + & \;6598820480576x^{14}+16634417823736x^{13}+39675706324196x^{12}+89928227622800x^{11}\\ + & \;194612460134000x^{10}+401859023378792x^{9}+788438707633360x^{8}+1461344582133568x^{7}\\ + & \;2538572309058668x^{6}+4082531612066208x^{5}+5933463106054116x^{4}+7526552803854528x^{3}\\ + & \;7900349393711252x^{2}+6259165196120000x+2805435842133989\\ R_{24}^{2}\left(x\right)= & \;16352800x^{24}+80984384x^{23}+1593580608x^{22}+8563164992x^{21}+49481490336x^{20}\\ + & \;209128258432x^{19}+780553489664x^{18}+2534712442464x^{17}+7427244572256x^{16}\\ + & \;20018378150080x^{15}+50545406056048x^{14}+120517158487296x^{13}+274250565071952x^{12}\\ + & \;596443605380944x^{11}+1241847578536336x^{10}+2470286333452336x^{9}+4675590805599856x^{8}\\ + & \;8364173069180016x^{7}+14021592964921728x^{6}+21731748892337248x^{5}+30399267731003712x^{4}\\ + & \;37067489321787104x^{3}+37349651746541568x^{2}+28288135205527104x+12068183577568336\\ R_{25}^{2}\left(x\right)= & \;13121920x^{26}+884160640x^{24}+5129076752x^{23}+31258322544x^{22}+163448687184x^{21}\\ + & \;673847529408x^{20}+2448076436168x^{19}+7870806586528x^{18}+22879203785480x^{17}\\ + & \;61395715879008x^{16}+154754196481972x^{15}+369113099563228x^{14}+840831639236328x^{13}\\ + & \;1836165518028176x^{12}+3848304176192976x^{11}+7732457069559076x^{10}+14866687805810788x^{9}\\ + & \;27211686368050264x^{8}+47088065652534764x^{7}+76324076542627132x^{6}\\ + & \;114226197524214276x^{5}+154090893839516976x^{4}+180963718118934524x^{3}\\ + & \;175352170716287904x^{2}+127221758063682128x+51780184823512125\\ R_{26}^{2}\left(x\right)= & \;4248992x^{28}+97321664x^{26}+2935299616x^{25}+16031112768x^{24}+107345361344x^{23}\\ + & \;515765014656x^{22}+2146804896608x^{21}+7657995466208x^{20}+24447054721696x^{19}\\ + & \;70731281782176x^{18}+189406512789664x^{17}+476321990810096x^{16}+1137098075502304x^{15}\\ + & \;2592711929684592x^{14}+5677359725456640x^{13}+11960396049047784x^{12}\\ + & \;24230629589854688x^{11}+47134110567515792x^{10}+87800322914710832x^{9}\\ + & \;155747494426559096x^{8}+261190652313599472x^{7}+410032082562386528x^{6}\\ + & \;593574085868314640x^{5}+773538638885231400x^{4}+876451928289172336x^{3}\\ + & \;818055164868369352x^{2}+569600084353206624x+221644202765881572 \end{array}$$

$$\begin{array}{cc} R_{27}^{2}\left(x\right)= & \;33298144x^{28}+1640322304x^{27}+9083937312x^{26}+66242344904x^{25}+379198633376x^{24}\\ + & \;1732710867064x^{23}+7010937195440x^{22}+24505702193136x^{21}+77097754182576x^{20}\\ + & \;221358499474104x^{19}+589907536190040x^{18}+1478844833406316x^{17}+3526024950737956x^{16}\\ + & \;8043136026455016x^{15}+17643778862079308x^{14}+37298778187795760x^{13}+76052804412338028x^{12}\\ + & \;149343565277026468x^{11}+281932903925939520x^{10}+509838659260734976x^{9}\\ + & \;878094236863577488x^{8}+1429425403496844584x^{7}+2176629333032060132x^{6}\\ + & \;3052538522347903156x^{5}+3848922745756731876x^{4}+4213944967774614108x^{3}\\ + & \;3794250917230376224x^{2}+2539731773644946096x+946672152353459593 \end{array}$$
